# A randomized, active-controlled, parallel, open-label, multicenter, phase 4 study to compare the efficacy and safety of pregabalin sustained release tablet and pregabalin immediate release capsule in type II diabetic patients with peripheral neuropathic pain

**DOI:** 10.1097/MD.0000000000033701

**Published:** 2023-04-28

**Authors:** Seon Mee Kang, Jun Hwa Hong, Bon Jeong Ku

**Affiliations:** a Department of Internal Medicine, Kangwon National University Hospital, Kangwon National University School of Medicine, Chuncheon, South Korea; b Department of Internal Medicine, Eulji University Hospital, Eulji University School of Medicine, Daejeon, South Korea; c Department of Internal Medicine, Chungnam National University Hospital, Chungnam National University School of Medicine, Daejeon, South Korea.

**Keywords:** analgesics, diabetic neuropathies, medication adherence, pregabalin

## Abstract

**Background:**

Diabetic peripheral polyneuropathy is the most common chronic complication of type 2 diabetes. Neuropathic pain is challenging to manage, and various drugs are required to control it, decreasing treatment adherence. Pregabalin, a ligand that binds to alpha-2-delta subunits of the presynaptic calcium channel, has been approved by the Food and Drug Administration for the treatment of diabetic neuropathic pain. In this study, we will compare the efficacy, safety, treatment satisfaction, and compliance between pregabalin sustained-release (SR) tablets and pregabalin immediate-release (IR) capsules in type 2 diabetic patients with peripheral neuropathic pain.

**Methods:**

This study is a randomized, active-controlled, parallel, open-label, multicenter, phase 4 clinical trial (trial registration NCT05624853). Type 2 diabetic patients with glycosylated hemoglobin below 10% and peripheral neuropathic pain who have been taking pregabalin 150 mg/d or more for more than 4 weeks will be randomly assigned to pregabalin SR tablet (150 mg once a day, n = 65) or pregabalin IR capsule (75 mg twice a day, n = 65) therapy for 8 weeks. The primary outcome will be the efficacy of SR pregabalin after 8 weeks of treatment, which will be assessed by visual analog scale measurements. The secondary outcomes will include changes in several parameters, such as quality of life, treatment satisfaction, quality of sleep, and drug compliance.

**Discussion:**

In thus study, we aim to demonstrate that pregabalin SR tablets are associated with better compliance and satisfaction compared with pregabalin IR capsules, despite similar efficacy.

## 1. Introduction

Diabetic neuropathy is the most prevalent chronic complication of type 1 and 2 diabetes mellitus; over 50% of people living with diabetes will develop this condition throughout their lifetime.^[1–3]^ Among the broad spectrum of diabetic neuropathies, diabetic peripheral neuropathy (DPN) is the most common type. Neuropathic pain, a major problem in DPN, remains difficult to manage despite previous efforts to develop therapeutic approaches. This results in several problems such as sleep disturbances, reduced quality of life, and socioeconomic consequences (e.g., depressed mood, reduced ability to work or perform daily activities, and increased health care costs).^[4–6]^ Painful DPN can eventually lead to a loss of sensation and lower limb amputation, which can increase mortality; therefore, early diagnosis and treatment are critical.^[7]^

Pregabalin, a ligand of the alpha-2-delta subunits of the presynaptic neuronal calcium channel, reduces pain by inhibiting the release of neurotransmitters involved in pain sensation.^[2,8]^ This drug was approved by the U.S. Food and Drug Administration in 2004. It is indicated for the treatment of neuropathic pain associated with DPN, spinal cord injury, and postherpetic neuralgia,^[8,9]^ and is generally considered a first-line treatment for painful DPN in most guidelines.^[10]^ There is evidence that the efficacy of pregabalin against painful DPN is dose-dependent.^[1,11–13]^ It can be administered twice or occasionally 3 times daily, and most patients require 300 mg/d or more for symptomatic relief. Recently, an extended-release formulation of pregabalin was approved by the U.S. Food and Drug Administration for the treatment of painful DPN and postherpetic neuralgia.^[14]^ Additionally, a new, once-daily, sustained-released (SR) pregabalin formulation (YHD1119 tablets; Yuhan Corporation, Seoul, Republic of Korea; now marketed as Yuhan Pregabalin SR Tab.) was shown to be effective and safe in a randomized, non-inferiority phase 3 study conducted in Korea.^[15]^ However, there is limited real-world data comparing the efficacy, safety, and medication adherence between immediate-release (IR) and SR formulations of pregabalin. Therefore, in this study, we aim to evaluate the efficacy, safety, and medication adherence of pregabalin SR compared with those of pregabalin IR.

## 2. Methods

### 2.1. Study design

This is a randomized, active-controlled, parallel, open-label, multicenter, phase 4, non-inferiority study conducted at 8 sites using either pregabalin SR tablets or pregabalin IR capsules. This study has been registered at Clinical Trial.gov (identifier NCT05624853).

### 2.2. Study ethics

The study will be conducted in accordance with the Declaration of Helsinki and Ethical Guidelines for Clinical Research, and the institutional review boards at each site will review the protocol and other relevant documents prior to study initiation. All participants will be required to provide written informed consent before enrollment.

### 2.3. Study population

Patients with type 2 diabetes taking pregabalin IR 150 mg/d for at least 4 weeks before screening will be recruited. The inclusion and exclusion criteria are provided below:

• Inclusion criteria:
  1. Patients capable of providing written informed consent
  2. Male or female patients between 19 and 75 years old
  3. Patients with type 2 diabetes mellitus (T2DM) and glycosylated hemoglobin (HbA1c) ≤ 10%
  4. Patients who experience moderate to severe pain associated with DPN (visual analogue scale [VAS] score ≥ 30 at screening)
  5. Patients with stable concomitant treatment for underlying disease
  6. For women of childbearing potential:
    a. Negative serum or urine pregnancy test at the screening visit
    b. Willing to use appropriate contraception during the study
• Exclusion criteria:
  1. Patients who are hypersensitive to the study drug or any of its components
  2. Patients who are taking anticonvulsants
  3. Patients who are experiencing non-DPN-related pain
  4. Patients with other types of neuropathies not related to DPN
  5. Patients with abnormal clinical research laboratory results at screening, defined as:
    a. Estimated glomerular filtration rate < 30 mL/min/1.73 m^2^
    b. More than 3 times the upper normal limits of alanine transaminase or aspartate transaminase
  6. Patients with active liver disease
  7. Patients with a history of alcohol or drug abuse
  8. Patients determined by the investigator to be unsuitable for participation because of severe depression, uncontrolled mood disorder, or behavioral changes
  9. Pregnant or lactating women
  10. Patients participating in another clinical trial within 30 days of screening
  11. Patients deemed ineligible for this clinical trial by the investigator

### 2.4. Randomization and treatment

**Figure 1.**
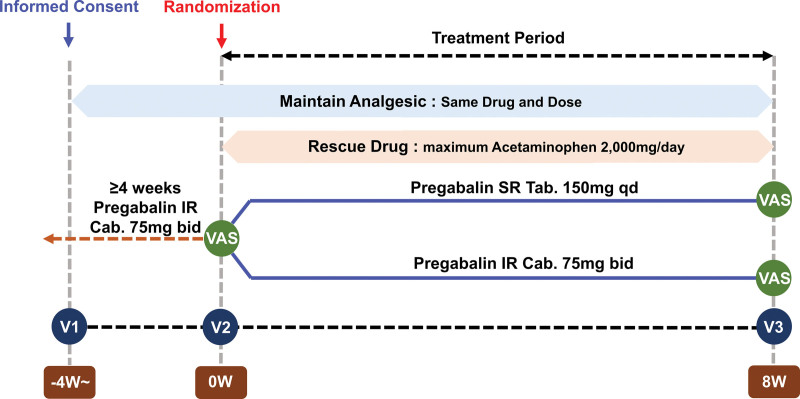
Study design.

Eligible participants who have VAS scores ≥ 30 at screening will be randomized to receive either IR or SR pregabalin in a 1:1 ratio by a computer-generated sequence without stratification. Pregabalin will be administered open-label, and patients will receive the assigned medication for 8 weeks. The study group will receive SR pregabalin tablets of 150 mg once a day starting in the evening, and the control group will receive IR pregabalin capsules of 75 mg twice a day until the morning of the last day of the study (Fig. 1). No dose adjustments will be made during the intervention period. If intolerable pain occurs during the trial, patients will be allowed to use rescue medication using acetaminophen at a maximum dose of 2000 mg/d. From 4 weeks before randomization until the end of the study, patients may use analgesics other than pregabalin as long as the same formulation and doses are maintained.

The investigator or clinical research coordinator will provide participants with instructions on how to take medication correctly. We will assess medication adherence by determining the amount of study drug not administered at the end of study visit (visit 3). At the final visit, all study drug (leftover tablets and packs) will be collected, and the investigator will check the quantity of the study drug. The number of days between the last visit and end of the treatment period will also be recorded. The percentage of medication adherence will be calculated by dividing the number of pills taken by the number of pills scheduled for administration during the treatment period. If a subject loses their study drug, they must receive a medical examination to confirm the loss, which will then be recorded in the source document and case report form.

### 2.5. Study endpoints and assessments

#### 2.5.1. Primary endpoint.

The primary endpoint of this study will be changes in the VAS score, defined as the difference in VAS scores from the start of the study and after 8 weeks of treatment.

#### 2.5.2. Secondary endpoints.

The secondary endpoints will include the following:

1. Changes from baseline after 8 weeks of treatment in:
  a. Quality of life (EQ-5D-3L)^[16]^
  b. Treatment satisfaction of clinical subjects (Patient Global Impression of Change)
  c. Treatment satisfaction of the investigator (Clinician Global Impression of Change)
  d. Sleep disturbance (Daily Sleep Interference Scale)^[17]^
  e. Medication adherence index (Morisky medication adherence scale 8-item version)^[18]^
2. Frequency, dose, and duration of rescue medication use after administration of the study drug

#### 2.5.3. Safety and clinical laboratory tests.

Height and weight will be recorded at the first visit. Smoking history, alcohol consumption, and vital signs will be assessed at each visit. Clinical laboratory tests will include complete blood count and measurement of aspartate aminotransferase, alanine aminotransferase, alkaline phosphatase, creatinine kinase, uric acid, total bilirubin, total protein, albumin, sodium, potassium, chloride, phosphorus, blood urea nitrogen, serum creatinine, and HbA1c levels. Biomarkers will be measured from a blood sample from each patient at each visit, with the exception of HbA1c. The estimated glomerular filtration rate will be calculated using the creatinine-based Modification of Diet in Renal Disease equation.^[19]^ The number of subjects, incidence rate and number of occurrences of treatment-emergent adverse events, adverse drug reactions, and serious adverse events after pregabalin administration will be recorded and analyzed.

### 2.6. Statistical analysis

Based on previous studies of IR pregabalin compared with placebo in patients with DPN,^[20]^ the sample size will be calculated as follows: with a margin of non-inferiority of 9.2 mm, 80% statistical power, and a significance level of 2.5% for 1-sided testing, assuming no difference in VAS scores between the 2 groups and a standard deviation of 0.6, approximately 65 subjects per treatment group will be required to demonstrate non-inferiority. Assuming a 20% dropout rate, 130 participants will be recruited (65 per group).

Full Analysis Set will be used as the main analysis group to evaluate efficacy while Per-Protocol Set will be used as a subanalysis set; Safety Set will be analyzed for safety data.

Differences in VAS scores in each group after 8 weeks of treatment compared with baseline will be described statistically (number of subjects, mean, standard deviation, median, minimum, and maximum) and analyzed by analysis of covariance corrected for baseline as a covariate. SR pregabalin will be considered non-inferior to IR pregabalin if the lower bound of the 95% confidence interval for the least squares mean difference between the treatment groups is greater than the prespecified non-inferiority margin of 9.2 mm. The secondary endpoints will be analyzed using either analysis of covariance or Pearson’s chi-square test, depending on the characteristics of the variables. In addition, demographics, baseline characteristics, treatment compliance, and safety endpoints will be summarized using descriptive statistics. Treatment adherence will be calculated by dividing the number of tablets taken by the number of tablets scheduled for administration and then multiplying by 100. The data will be analyzed using IBM SPSS Statistics for Windows, Version 20 (IBM, Armonk, NY).

## 3. Discussion

We will evaluate the real-world efficacy and safety of SR pregabalin and compare medication adherence among T2DM patients with painful DPN. The SR pregabalin formulation has been developed using a floating and swelling gastro-retentive drug delivery system to prolong the gastric retention of pregabalin, and it can be administered once a day.^[21]^ In a previous randomized double-blind study in South Korean patients with DPN and postherpetic neuralgia, the efficacy of the new SR pregabalin (YHD1119), as measured by the mean Daily Pain Rating Scale score, was non-inferior to IR pregabalin.^[15]^ However, there was no assessment of drug compliance when the IR formulation is switched to the SR formulation from twice-daily to once-daily dosing.

People with chronic conditions, such as cardiovascular disease and diabetes, are frequently confronted with the need to regularly take a substantial number of medications over extended periods of time owing to their underlying disease and other comorbidities.^[22]^ This high pill burden contributes to poor medication adherence, resulting in practice/outcome gaps that limit the expected therapeutic benefits.^[23]^ Previous studies have shown that reducing the frequency of dosing in patients with chronic conditions may enhance medication adherence and clinical outcomes. Therefore, our study aims to evaluate patient adherence to SR pregabalin versus IR pregabalin.^[24]^ In addition, we will evaluate treatment satisfaction from both the patient and physician perspectives after 8 weeks of SR pregabalin therapy. In summary, this randomized, active-controlled, parallel, open-label, multicenter, phase 4 study aims to confirm the real-world efficacy and safety of SR pregabalin in T2DM patients with painful DPN, in addition to evaluate medication adherence.

## Acknowledgments

The authors thank all participants in this trial.

## Author contributions

**Conceptualization:** Seon Mee Kang, Jun Hwa Hong, Bon Jeong Ku.

**Data curation:** Seon Mee Kang, Bon Jeong Ku.

**Formal analysis:** Bon Jeong Ku.

**Funding acquisition:** Bon Jeong Ku.

**Investigation:** Bon Jeong Ku.

**Methodology:** Seon Mee Kang, Jun Hwa Hong, Bon Jeong Ku.

**Project administration:** Bon Jeong Ku.

**Supervision:** Bon Jeong Ku.

**Visualization:** Seon Mee Kang, Bon Jeong Ku.

**Writing – original draft:** Seon Mee Kang, Jun Hwa Hong, Bon Jeong Ku.

**Writing – review & editing:** Seon Mee Kang, Jun Hwa Hong, Bon Jeong Ku.
